# Multiparametric radiobiological assays show that variation of X-ray energy strongly impacts relative biological effectiveness: comparison between 220 kV and 4 MV

**DOI:** 10.1038/s41598-019-50908-4

**Published:** 2019-10-04

**Authors:** Vincent Paget, Mariam Ben Kacem, Morgane Dos Santos, Mohamed A. Benadjaoud, Frédéric Soysouvanh, Valérie Buard, Tarlet Georges, Aurélie Vaurijoux, Gaëtan Gruel, Agnès François, Olivier Guipaud, Fabien Milliat

**Affiliations:** 1 Institute for Radiological Protection and Nuclear Safety (IRSN), Department of RAdiobiology and regenerative MEDicine (SERAMED), Laboratory of MEDical Radiobiology (LRMed), Fontenay-aux-Roses, 92260 France; 2 Institute for Radiological Protection and Nuclear Safety (IRSN), Department of RAdiobiology and regenerative MEDicine (SERAMED), Laboratory of Radiobiology of Accidental exposures (LRAcc), Fontenay-aux-Roses, France; 3 Institute for Radiological Protection and Nuclear Safety (IRSN), Department of RAdiobiology and regenerative MEDicine (SERAMED), Fontenay-aux-Roses, France

**Keywords:** Cell division, Cell death, Senescence

## Abstract

Based on classic clonogenic assay, it is accepted by the scientific community that, whatever the energy, the relative biological effectiveness of X-rays is equal to 1. However, although X-ray beams are widely used in diagnosis, interventional medicine and radiotherapy, comparisons of their energies are scarce. We therefore assessed *in vitro* the effects of low- and high-energy X-rays using Human umbilical vein endothelial cells (HUVECs) by performing clonogenic assay, measuring viability/mortality, counting γ-H2AX foci, studying cell proliferation and cellular senescence by flow cytometry and by performing gene analysis on custom arrays. Taken together, excepted for γ-H2AX foci counts, these experiments systematically show more adverse effects of high energy X-rays, while the relative biological effectiveness of photons is around 1, whatever the quality of the X-ray beam. These results strongly suggest that multiparametric analysis should be considered in support of clonogenic assay.

## Introduction

Relative biological effectiveness (RBE) is the ratio of biological effectiveness of one kind of ionizing radiation relative to another. Several studies have focused on X-rays and their effects upon cells, but few have directly compared two different X-ray beams. Moreover, there is a consensus that the RBE of X-rays (photons; energy from 0.1 to 3 MeV) is equal to 1, whatever the energy of the beam^1^. RBE measurements are essentially based on clonogenic assay^2^, which is considered the gold standard. This assay is based on the ability of a single cell to grow into a colony after a stress^3–5^. Representation of the survival fraction (SF) as a function of the dose leads to survival curves, which are modeled by the linear-quadratic model (LQ-model)^6^. Despite these considerations, we think that clonogenic assay should be strengthened by other biological measurements to improve the prediction of cellular outcome after exposure to ionizing radiation. Interestingly, some authors also support the fact that this LQ-model is inappropriate for high dose per fraction^7^.

To validate this proof of concept, we exposed human normal endothelial cells (HUVECs) to low- and high-energy X-rays at the same doses and dose rates. HUVECs were chosen as a biological model due to their capability to form clones in dishes^8,9^ and because vascular injury is one of the most common effects of radiotherapy on normal tissues and tumors^10,11^. Finally, based on work currently in progress in our group (data submitted elsewhere in literature), HUVECs are the ones which have the higher response to irradiation among several other human primary endothelial cells (Human Pulmonary Artery Endothelial Cells (HPAEC), Human Dermal Microvascular Endothelial Cells (HMVEC-D), Human Intestinal Microvascular Endothelial Cells (HIMEC), Human Lung Microvascular Endothelial Cells (HMVEC-L) or Human Cardiac Microvascular Endothelial Cells (HMVEC-C)). Irradiation at 220 kV using our Small Animal Radiation Research Platform (SARRP, Xstrahl) was chosen as it is considered as equivalent to the classic beam references such as ^60^Co γ rays^12^. In order to be relevant to beams classically used in radiotherapy, the Linear Accelerator (LINAC) Elekta Synergy Platform (ELEKTA S.A.S. France, Boulogne, France) delivering 4 MV X-rays was chosen to assess the effects of high-energy X-rays *in vitro*.

HUVECs were irradiated with high- (4 MV) or low- (220 kV) energy X-rays in clonal conditions (clonogenic assay) and at confluence for all other assays (viability/mortality, γ-H2AX foci, cell proliferation and senescence). The overall results clearly indicate that, excepted for H2AX foci up to 10 hours post-irradiation, high-energy X-rays significantly induce more adverse effects in HUVECs than low-energy X-rays. Our findings clearly show that the RBE of X-rays (energy from 0.1 to 3 MeV) is not equal to 1.

## Results

### Clonogenic assay and cell viability

**Figure 1:**
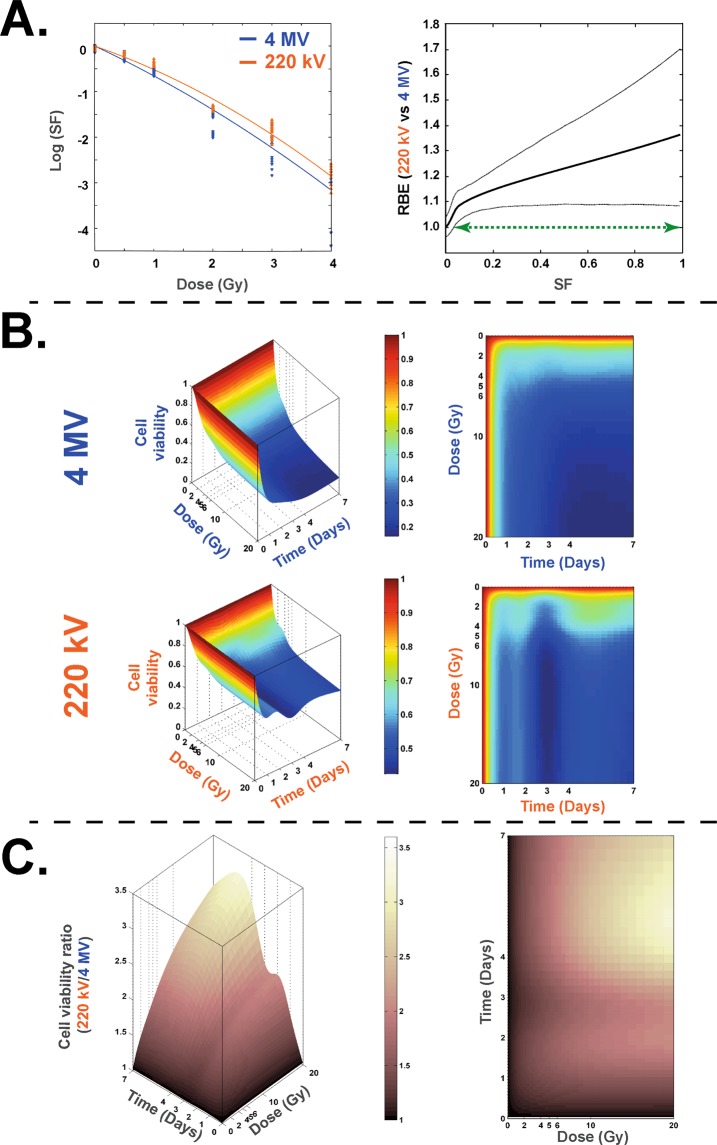
Clonogenic assay and cell survival at 220 kV and 4 MV. (**A**) left panel: Survival fraction (SF) of HUVECs irradiated at 220 kV (orange curve) and 4 MV (blue curve). Right panel: the associated RBE curve defined as a ratio of doses for a given SF (solid line) and its associated bootstrap confidence intervals (dotted lines). The green arrow representing the range of SF wherein the value of RBE is significantly different from 1. (**B**) Representations of 3D and 2D curves of cell survival measures with trypan blue counting method for 220 kV and 4 MV. (**C**) Representations of 3D and 2D curves of cell viability ratio [220 kV/4 MV].

Cell survival curves show a classic dose-dependent decrease of the survival fraction for both irradiations (Fig. 1A). Furthermore, the statistical analysis method used^9^ and the RBE calculation (ratio of doses 220 kV/4 MV for a given SF) has shown a significant difference between the two kinds of irradiation (green curve in right panel on Fig. 1A), numerical values of calculated RBE being reported on Supplementary Table S1. These results were corroborated by cell viability counting using the trypan blue method (Fig. 1B), showing higher cell viability after irradiation at 220 kV compared to 4 MV. A statistical representation of 220 kV/4 MV cell viability is shown in Fig. 1C, showing that, whatever the time or the dose, cell viability was significantly higher at 220 kV compared to 4 MV irradiation. A “RBE” calculation (ratios for a given dose and time between the two beams) is reported on Supplementary Fig. S1, showing a wide range of values depending on the end-point.

### DNA double-strand break measurements (γ-H2AX foci)

**Figure 2:**
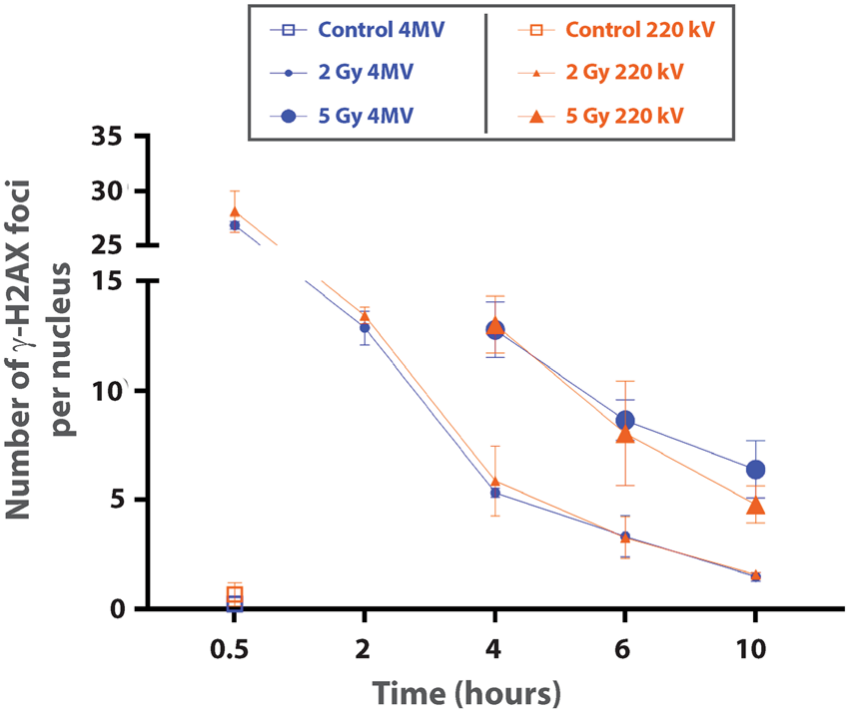
DNA double-strand break measurements (γ-H2Ax foci). Number of γ-H2AX foci in G0/G1 primary HUVECs as a function of time since exposure. The analyses were performed on cells exposed to irradiation with 4 MV (blue curves) and 220 kV (orange curves) beams. Mean number of γ-H2AX foci per nucleus for non-irradiated cells at 30 min are represented by squares, small symbols correspond to the mean number of γ-H2AX foci per nucleus for the 2 Gy dose and large symbols correspond to 5 Gy irradiation. The mean ± standard deviation (s.d.) was calculated from 2 independent experiments. For each condition, the average number of analyzed cells was around 600.

We ensured that HUVECs were at around 80% in G1 phase (Supplementary Fig. S2) before 4 MV or 220 kV irradiation. Whatever the modality of irradiation, we observed, from 30 min to 10 h post-irradiation, a classic decrease of the number of γ-H2Ax foci per nucleus over time (Fig. 2). Nevertheless, no significant difference in γ-H2AX foci number per nucleus was observed between the two kinds of irradiation for the same end-point/dose (Fig. 2).

### Proliferation (Click-iT Technology) (Flow Cytometry)

**Figure 3:**
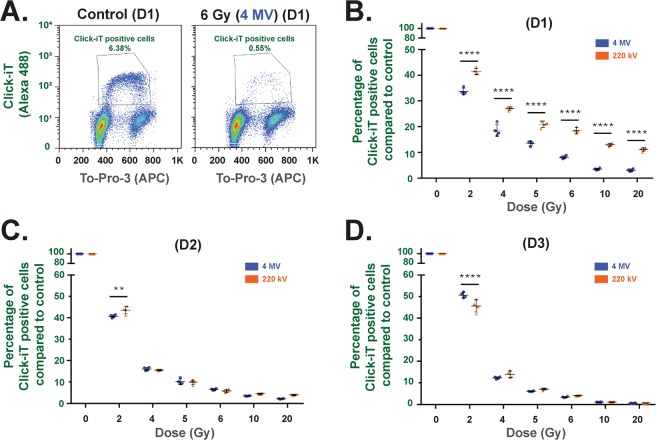
Proliferation measurements using Click-iT technology. (**A**) Example of flow cytometry measurements obtained at day 1 for control and 6 Gy irradiation at 4 MV. (**B** to **D**) Percentage of Click-iT positive cells for 220 kV and 4 MV irradiations at day 1 (**B**), day 2 (**C**) and day 3 (**D**) post-exposure. Each dot represents one independent experiment (**p < 0.01, ****p < 0.001, two-way Anova).

Analysis set-up for Click-iT EdU experiments are described in Supplementary Figs S3 and S4. Proliferation experiments (Fig. 3A.) showed that, at 24 hours (D1) post-irradiation, whatever the dose, a significantly (p < 0.0001) higher percentage of EdU-positive cells was obtained at 220 kV compared to 4 MV (Fig. 3B). At 48 (D2) and 72 (D3) hours post-irradiation (Fig. 3C,D), the percentage of EdU-positive cells was not significantly different between the two energies, except at the dose of 2 Gy.

### Cell Division (CellTrace Technology)/ Senescence (C_12_FDG) (flow cytometry)

**Figure 4:**
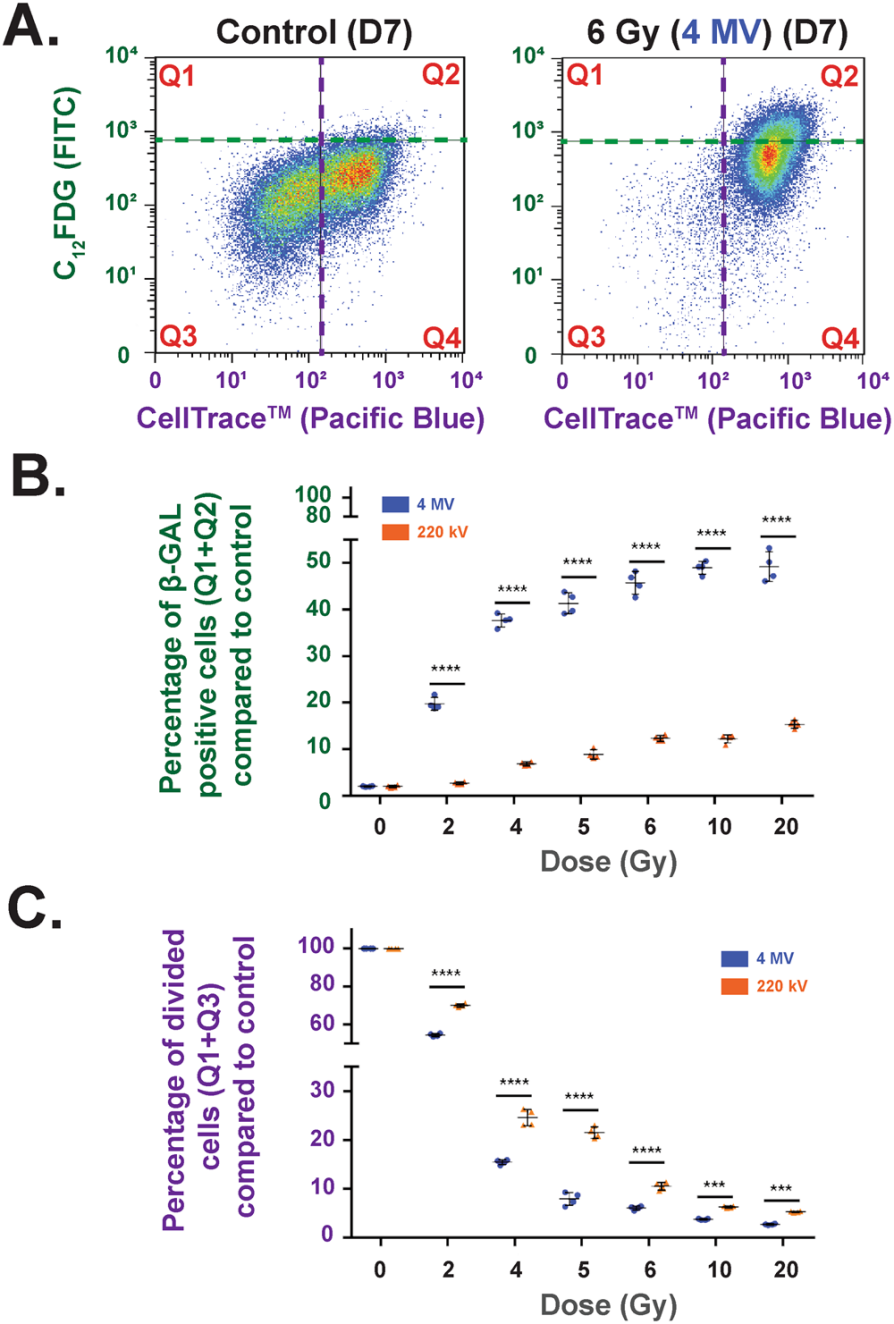
Cell Division (CellTrace Technology)/ Senescence (C_12_FDG) (Flow Cytometry) bi-parametric analysis (A) Example of flow cytometry measurements obtained at D7 for control and 6 Gy irradiation at 4 MV. (**B**) Percentage of β-GAL-positive cells (Q1 + Q2) compared to control at 4 MV (blue dots) and 220 kV (orange dots) irradiations. Each dot represents one independent experiment (****p < 0.001, one-way Anova). (**C**) Percentage of divided cells (Q1 + Q3) compared to control for 220 kV (orange dots) and 4 MV (blue dots). Each dot represents one independent experiment (***p < 0.001, ****p < 0.001, one-way Anova).

5-bromo-4-chloro-3-indolyl-β-D-galactopyranoside (X-GAL) staining of HUVECs 7 days after 20 Gy irradiation at 4 MV was performed to ensure that senescence was induced after irradiation (Supplementary Fig. S5). Thus, analysis set-up for flow cytometry CellTrace/C_12_FDG experiments is described in Supplementary Fig. S6. This bi-parametric approach was chosen, as senescent cells are metabolically active but unable to divide. Our experiments demonstrate that when the dose increases, the number of senescent cells increases (Fig. 4A,B) compared to control and the number of divided cells decreases as well (Fig. 4C), whatever the energy of the beam. Nevertheless, our results systematically show, for each dose, a significant difference between 4 MV and 220 kV irradiations. Indeed, whatever the dose, more senescent cells (Fig. 4B) and fewer divided cells (Fig. 4C) were obtained after 4 MV than 220 kV irradiation.

### RT-qPCR (customized Taqman Low-Density Assay [TLDA])

**Figure 5:**
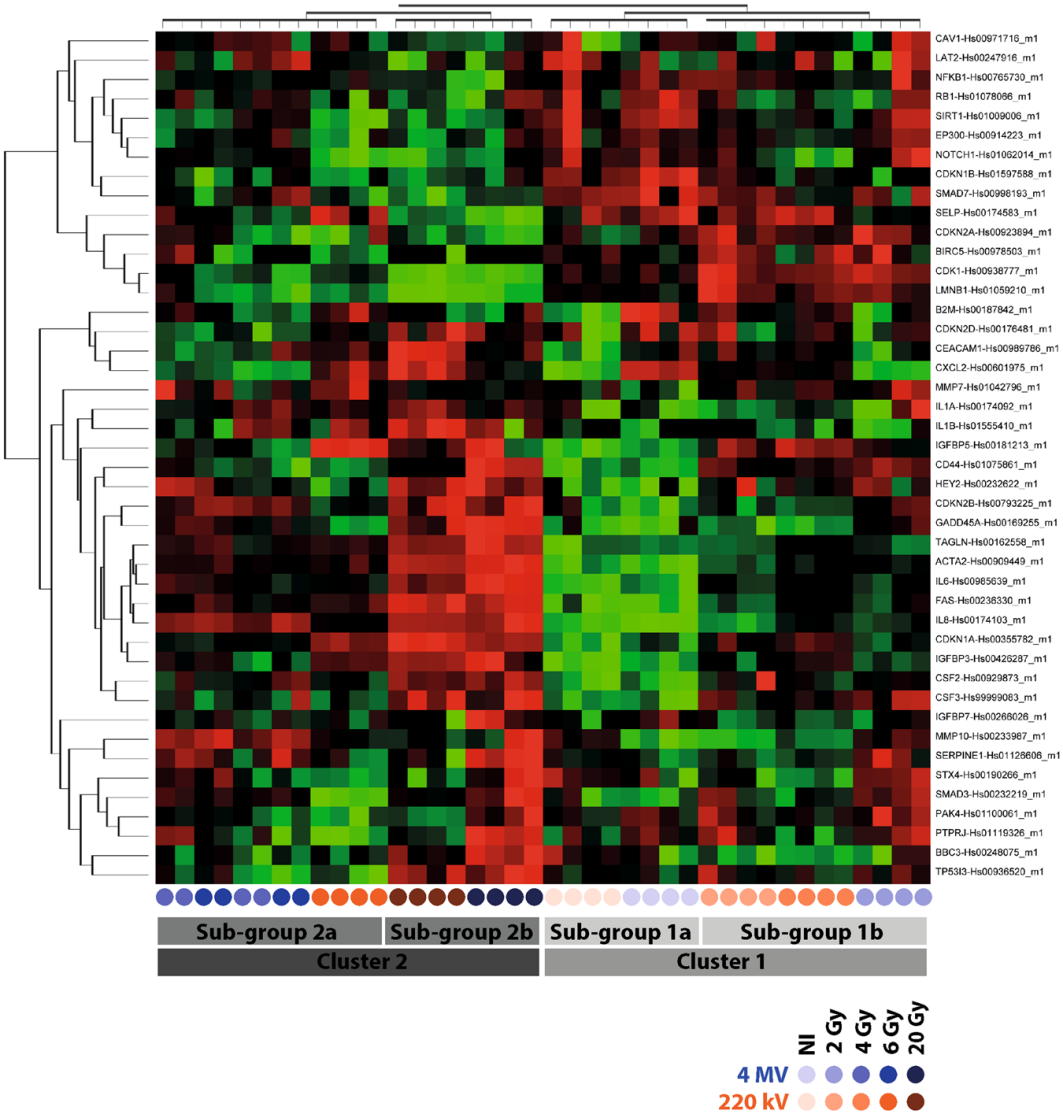
RT-qPCR. Assay heat map illustrating differentially expressed genes among 44 genes measured at day 7 post-irradiation. For one gene considered, red dots represent overexpression and green dots represent downregulation. Corrected p-values using the Benjamini-Hochberg method, p < 0.05. (n = 4 independent experiments per condition). Blue rounds correspond to 4 MV and orange rounds to 220 kV, the color gradient indicating the dose.

Unsupervised hierarchic clustering showed significant differences between the two kinds of irradiations (Fig. 5). Two big clusters were identified. The first one, named “cluster 1”, contains all controls and 2 Gy plus 4 Gy at 220 kV. Within this “cluster 1”, we also have two sub-groups, both controls on the one hand (“sub-group 1a”), and on the other hand both 2 Gy plus 4 Gy at 220 kV (“sub-group 1b”). The second big cluster, called “cluster 2”, hosts all other conditions and is also divided in two sub-groups: “sub-group 2a” which contains 4 Gy at 4 MV and both 6 Gy conditions, and “sub-group 2b” which hosts both 20 Gy conditions. As an example of genes analysis, IL-8, IL-6 and MMP10 which are ones of the key markers of the SASP, were significantly increased with the dose 7 days post-irradiation (Supplementary Fig. S7) for both beams.

## Discussion

To date, no extensive study has compared two beams of X-rays with high and low energy at the same dose rate or has combined a wide range of biological outputs. Only few studies have reported a higher RBE for X-rays compared to the reference beam (200–250 kV)^12–15^. Nevertheless, these studies were performed on very low energy X-rays (below 50 kV) and not necessarily at the same dose rate than the reference X-rays beam. Furthermore, RBE can be determined by using different endpoints such as clinical outcomes but are essentially based on proton beams in hadron therapy^16–18^. Thus, our study focused on cellular outcome up to 7 days after 220 kV and 4 MV X-ray irradiation, using the same dose rate (2.5 Gy/min), and covering a wide range of doses (0, 2, 4, 5, 6, 10 and 20 Gy).

Our statistical analysis of the clonogenic assay shows that for the whole range of doses tested (0 to 4 Gy) the two survival curves are significantly different (indicated by green arrow in Fig. 1A, right panel). Referring to the classic SF 2 Gy^19^, we obtained an RBE around 1.246 (220 kV/4 MV). When irradiation was performed on a confluent HUVEC monolayer, the modelling of cell survival (Fig. 1B) differed significantly over time and over the whole range of doses between the two modalities of irradiation, testifying to the greater deleterious effect in the cell population when the X-ray energy increases (Fig. 1C). Interestingly, it is now known that exposure to very low-energy X-rays (25–30 kV) leads to high levels of DNA damage^12^ or micronuclei^15^. But, surprisingly, our two beams are within the range of energy from 0.1 to 3 MeV for which an RBE of 1 is classically ascribed^1^.

In order to validate these findings, we extended the work to other biological output measurements. DNA damage was measured at the same time according to the distribution of the number of γ-H2AX foci per nucleus (Fig. 2). The H2AX method was chosen as it is very sensitive in detecting DNA double-strand breaks^20^ and predicts *in vivo* genotoxicity^21^. As the cell cycle phase can strongly affect the results after irradiation^22^, we ensured that HUVECs were at around 80% in G1 phase (Supplementary Fig. S2) before 4 MV or 220 kV irradiation. This state of confluency mimics a synchronized cell population without performing serum depletion, which is known to induce cell death, depending on the cell type. Regarding the number of γ-H2AX foci per nucleus from 30 min to 10 h post-irradiation at the doses of 2 and 5 Gy, we found no significant difference between the two kinds of beams (Fig. 2). Even though the mean number of γ-H2AX foci per nucleus classically decreases over time, Click-iT experiments 6 hours post-irradiation at 4 MV (Supplementary Fig. S4) showed that incorporation of EdU is strongly altered for doses above 6 Gy. This may suggest that complex damage is induced and is not only based on DNA double-strand breaks. Moreover, it would be interesting to further investigate oxidative stress induced by both beams by measuring reactive oxygen species (ROS) with a CM-H_2_DCFDA probe^23^ or by glutathione depletion. Also, mitochondrial dysfunction could be another trail to investigate, in order potentially to shed light on differences between the two kinds of beams. Such a phenomenon has been reported after exposure to ionizing radiation^24^ and, more particularly, in human endothelial cells from lung^25^.

Radiation-induced senescence is now well described and is characterized by an increase of cell size and β-galactosidase activity^26^. It has been hypothesized that induction of senescence by ionizing radiation not only mediates the ignition of pulmonary fibrosis, but also plays a critical role in the progression of this disease^27^. To verify radiation-induced senescence *in vitro* in HUVECs, we performed staining with X-GAL, a widely used biomarker^28,29^. As reported by Debacq-Chainaux^29^, we used bafilomycin A1 pre-treatment of the samples to be more specific to β-galactosidase activity linked with stress-induced senescence. X-GAL staining of HUVECs 7 days after 20 Gy irradiation at 4 MV (Supplementary Fig. S5) was strong, corroborating the literature data^30^. Furthermore, staining was localized on accumulated lysosomes within enlarged cells with more flattened morphology, which are characteristics of senescent cells as already reported in the literature^26^. To compare radiation-induced senescence for the two beams, we used flow cytometry with C_12_FDG instead of X-GAL staining. By fluorescence measurement within the cell, C_12_FDG staining i) is very sensitive for a very large number of events, and ii) is a representative response of the whole cell monolayer^29^. Moreover, senescent cells are blocked in the cell cycle^31^, but remain metabolically active. Interestingly, we have observed that at higher doses, fewer cells are able to re-enter division after irradiation (Fig. 3). Thus, our data fully corroborate the phenomenon recently reported by Reyes *et al*.^32^, who have demonstrated that fluctuations in p53 signaling allow escape from cell-cycle arrest, which we also observed 7 days after irradiation when the population of “divided cells” (Q1 + Q3) decreased according to the dose (Fig. 4). With these two approaches, we have shown that after irradiation the cellular outcome is driven to undivided and positive β-galactosidase cells, two markers of senescence. Whatever the beam, the percentage of senescent cells increases according to the dose, but remains significantly higher after 4 MV irradiation compared to 220 kV.

Finally, we performed gene analysis by custom TLDA of 44 genes reported to be involved in the senescence process and the senescence-associated secretory phenotype (SASP)^26,31,33^. The unsupervised hierarchical clustering performed on custom TLDA showed a macroscopic clustering on the dose rather on the energy (Fig. 5). Indeed, several genes (such as p16 (CDKN1A) or p21 (CDKN2A)) are very well-known to be deregulated by irradiation at delayed times. This could partly explain the macroscopic clustering according to the dose only seven days post-irradiation. Nevertheless, if we analyze more precisely key genes of the SASP (IL8, IL-6 and MMP10), we effectively found significant differences according to the energy (Supplementary Fig. S7A), which were not obvious on the heatmap clustering. Indeed, IL-8 and MMP10 were significantly increased with the dose 7 days post-irradiation (Supplementary Fig. S7A) for both beams. Furthermore, this upregulation was systematically higher at 4 MV than at 220 kV (Supplementary Fig. S7A), fully corroborating our C_12_FDG results. Furthermore, BIRC5, which encodes for survivin, an inhibitor of apoptosis, was not detected at 4 MV since the dose of 6 Gy (on 3 of 4 experiments) (Supplementary Fig. S7B). This perfectly corroborates our results on cell viability (Fig. 1), which reported a significant higher mortality at 4 MV compared to 220 kV. Finally, Cdk-1, a cyclin involved in the progression of phases S and G2 within cell cycle, was logically found decreased according to the dose for both beams; but this downregulation was systematically higher at 4 MV than at 220 kV (Supplementary Fig. S7C), fully corroborating our results of proliferation and senescence (Figs 3 and 4).

From a clinical point of view, modern radiotherapy also uses modifications of energy electron beam i.e. flattening-filter-free (FFF), thus assuming that biological effects are the same for a flattened beam. However, our current work using multiparametric biologic readouts is the proof of concept that the RBE of X-rays depends on the energy of the beam. This clearly raises the question as to whether or not, in radiotherapy, the biological effects of X-rays for normal tissues and/or tumors are the same, whatever the energy of the beam.

In conclusion, we observed more adverse effects in HUVECs after 4 MV compared to 220 kV X-rays, clearly establishing that the RBE for X-rays of different energies is not equal to 1 and can vary strongly depending on the assay. Furthermore, these multiparametric assays can provide an answer in the comparison of two beams in the case of high doses (i.e. doses for which a clonogenic assay cannot be performed). Such an approach could also be useful in comparing the RBE with dose rate modifications (in connection or not with energy modification), as conventional radiobiological models also fix the RBE at 1.

## Methods and Materials

### Irradiation facilities: SARRP and LINAC

Irradiation dosimetry and the 220 kV protocol are already described in the literature^9^. To avoid dosimetry errors and to keep X-ray field homogeneity, only 4 of 6 wells were used for *in vitro* experiments on the SARRP platform^9^. Sterile thin films were used to replace plastic cover on plates during irradiation, to avoid any attenuation of the X-ray spectrum^9^. Irradiation with high-energy X-rays was performed using an Elekta Synergy Platform (ELEKTA S.A.S. France, Boulogne, France) delivering 4 MV X-rays. With both facilities (SARRP and LINAC), irradiations were performed under similar conditions: plate, cell culture medium and a dose rate of about 2.5 Gy/min in air kerma free in air. The uncertainty in the dose rate measurement was about 5% and 7% for SARRP and LINAC irradiations, respectively at k = 2.

### Cell culture

Human umbilical vein endothelial cells (HUVECs, C2519A) from LONZA were cultured in EGM-2 MV culture medium (LONZA) according to the manufacturer’s instructions and placed in an incubator at 37 °C with 5% CO_2_ and 95% humidity. For all the experiments, HUVECs at passage 2 were seeded at 3 × 10^3^ cells/cm^2^ and routinely cultured for 5 days to reach confluent monolayers. HUVECs were then detached and seeded (3 × 10^3^ cells/cm^2^, passage 3), and cultured for 5 days to reach confluent monolayers for all experiments (excluding the clonogenic assay where specific conditions are detailed in the corresponding section).

### Clonogenic assay

Cells were irradiated on LINAC or SARRP (0 (control), 0.5, 1, 2, 3 and 4 Gy) by following a protocol already described in the literature^9^. Briefly, cells were seeded in 6-well culture plates (1 × 10^3^ cells/well for doses below 2 Gy, 2 × 10^3^ cells/well for doses above 2 Gy) and, three hours after plating, were irradiated at different doses (0 (control), 0.5, 1, 2, 3 and 4 Gy). After this adhesion interval, microplates were irradiated on the SARRP at 220 kV (using additional 0.15 mm copper filtration) or at LINAC at 4 MV, both at the same dose rate of 2.5 Gy/min. Nine days after irradiation, cells were fixed for 15 min with 4% final (v/v) paraformaldehyde (in Phosphate-Buffered Saline (PBS) without Ca^2+^ and Mg^2+^) and then stained for 30 min with Giemsa (SIGMA ALDRICH) at a final concentration of 10% (v/v) (in milliQ water). Colonies containing more than 60 cells (corresponding to at least 6 doubling times)^4,9^ were counted.

### Viability/Mortality (trypan blue)

At each endpoint, supernatant was collected; cells were trypsinized and added to the respective supernatant. Each sample was centrifuged for 5 min at 200 g and the pellet was resuspended in 1 mL of PBS. Cells were manually counted under the microscope by using trypan blue (145-0013, BIO-RAD Laboratories S.A.) and 10-chambered slides with a hemocytometer-type grid (87144, KOVA Glasstic Slide 10 with Grids).

### DNA double-strand break measurements (γ-H2AX foci)

#### Immunofluorescence staining

Cells were fixed with 4% paraformaldehyde solution (199431LT, AFFYMETRIX), washed with 1X PBS (14190-094, THERMO FISHER SCIENTIFIC), permeabilized with 0.5% Triton X-100 (T8787, SIGMA ALDRICH) and then washed with 1X PBS. Antibodies were diluted in 1X PBS with 2% (w/v) BSA (bovine serum albumin; A9418, SIGMA ALDRICH). IgG1 monoclonal anti-phospho-histone H2AX (Ser139) antibody (dilution of 1/800; 05-636, clone JBW301, UPSTATE) was incubated with cells for 1 hour at room temperature (RT). Cells were then washed and incubated for 1 hour at RT with the secondary antibody goat anti-mouse IgG1 (γ1) coupled to Alexa Fluor 488 (2 mg.mL^−1^; A21121, THERMO FISHER SCIENTIFIC). After washing, DNA was stained with 4’,6-diamidino-2-phénylindole (DAPI) (0.2 µg.mL^−1^; 1050 A, EUROMEDEX) and mounted with ProLong Antifade Reagents (P36930, THERMO FISHER SCIENTIFIC).

#### Image acquisition and analysis

Images were acquired and analyzed with the Scan^R platform (OLYMPUS), as described previously^34,35^. Briefly, images were acquired on an inverted OLYMPUS IX81 fluorescence microscope with a UPLSAPO 100XO oil immersion objective (OLYMPUS) and an NA of 1.4; the microscope was coupled with an Orca R² CCD camera (HAMAMATSU) and a motorized SCAN IM IX2 stage (MARZHAUSER). An edge segmentation algorithm based on Canny’s method^36^ was used to detect nuclei in the DAPI channel (main object) and γ-H2AX foci in the FITC channel (sub-object 1). A first selection based on the area and circularity of the nuclei excluded clusters of cells and cellular debris. Cells were then selected in G0-G1 phase of the cell cycle by assessing the integrated intensity of the DAPI signal (DNA content) combined with the integrated intensity of the γ-H2AX signal in the entire nucleus, which increased dramatically in S phase^34,37^. Gamma-H2AX foci in the objects within the gate formed by the intersection of the two regions were then analyzed.

### Proliferation (Click-iT Technology) (Flow Cytometry)

Aphidicolin (5 µM final, SIGMA ALDRICH, ref A0781) was added to negative control conditions 3 hours before the endpoint. Then, two hour before the endpoint (24; 48 or 72 hours post-irradiation), EdU from Click-iT EdU Alexa Fluor 488 Flow Cytometry Assay Kit (C10420, THERMO FISHER SCIENTIFIC) was added to the cell culture medium at 10 µM final concentration. The monolayers of HUVECs were rinsed twice with PBS, cells were trypsinized, fixed/permeabilized and the Click-iT EdU Alexa Fluor 488 reaction was performed according to the manufacturer’s protocol. Finally, the pellet was resuspended in 1 mL of PBS before acquisition on a FACS Canto II. In order to avoid compensation, DNA was stained for 30 min at RT using To-Pro-3 (0.5 µg/mL final) and was recorded on the APC channel^38^. Acquisition of data was performed on a FACS Canto II (3-laser, 4-2-2 configuration) using FACS Diva software. Four independent experiments were performed for each condition. Data analysis was performed post-acquisition using FlowJo 7.6.5 software (FlowJo LLC). A first analysis was done on size (FSC: Forward scatter)/granulometry (SSC: Side scatter) parameters, to collect cells (gate G1) and to remove fragmented cells and debris (Supplementary Fig. S3, left row). The gated events (gate G1) were then plotted on an APC-A (intensity)/APC-W (size) graph in order to remove doublets and to perform analysis on the single events population determined by the gate G2 (Supplementary Fig. S3, middle row). Finally, events on gate G2 were plotted on an Alexa 488/APC-A dot plot to determine the percentage of EdU-positive cells (Gate G3) (Supplementary Fig. S3, right row). Analysis was performed on 4 independent experiments, where at least 5 × 10^4^ single cells per replica were recorded on gate G2. Acquisitions were performed using the following settings: DNA content was recorded on the APC channel (filters λ_em_: 660/20 nm) after 633 nm HeNe solid state (17 mW output) laser excitation, while EdU-positive cells were detected on the Alexa Fluor 488 channel (filters λ_em_: 530/30 nm) after air-cooled 488 nm solid state (20 mW output) laser excitation.

### Senescence (C12FDG)/Generation (CellTrace) (Flow Cytometry)

Before irradiation, confluent monolayers of HUVECs were stained using CellTrace violet (Ref C34557, THERMO FISHER SCIENTIFIC). Cells were rinsed twice with PBS 1 × (with Ca^2+^ and Mg^2+^), and then stained for 20 min with 7.5 µM final of CellTrace violet in PBS 1 × (with Ca^2+^ and Mg^2+^). After staining, the staining solution was removed and the monolayers were rinsed twice with fresh cell culture medium to neutralize excess CellTrace violet. Cells were then irradiated at 2, 4, 5, 6, 10 or 20 Gy + control (non-irradiated). Seven days after irradiation, senescence experiments were performed by following Debacq-Chainiaux *et al*.^29^ using 1-hour pre-treatment with bafilomycin A1 (100 nM final), followed by addition of C_12_FDG (33 µM final) for 2 hours. Supernatant was removed, monolayers were rinsed twice with PBS 1 × (without Ca^2+^ and Mg^2+^), cells were trypsinized and centrifuged for 5 min at 200 g and the pellet was resuspended in 1 mL of PBS before acquisition on a FACS Canto II. To increase the robustness of the results, a cell viability reporter was added to each sample: the To-Pro-3^38^ before the acquisition of the data on a FACS Canto II (3-laser, 4-2-2 configuration) using FACS Diva software, 4 independent experiments were performed for each condition. Data analysis was performed post-acquisition using FlowJo 7.6.5 software (FlowJo LLC). A first analysis was done on size (FSC: Forward scatter)/granulometry (SSC: Side scatter) parameters, to collect cells (gate G1) and to remove fragmented cells and debris. Triton 0.06X final was instantly used as positive control (Supplementary Fig. S6A) to ensure good detection of dead cells. This first step allowed us to assess cell viability (on the APC channel (filters λ_em_: 660/20 nm) after 633 nm HeNe solid state (17 mW output) laser excitation) and to determine the gate (G2) where at least 5 × 10^4^ living cells per replica were recorded (Supplementary Fig. S6B,C). Then, upon this gated event (Supplementary Fig. S6B,C), the C_12_FDG signal was collected on the FITC channel (filters λ_em_: 530/30 nm) after air-cooled 488 nm solid state (20 mW output) laser excitation, while the CellTrace violet signal was collected on the Pacific Blue channel (filters λ_em_: 450/50 nm) after 405 nm solid state (30 mW fiber power output) diode excitation. Combining CellTrace violet and C_12_FDG measurements, a bi-parametric representation was then possible and distinguished two kinds of populations: senescent cells called β-GAL + cells (Q1 + Q2) where a cut-off was set at 2% in control cells (Supplementary Fig. S6D), and “divided cells” called CellTrace - cells where the cut-off was set between the two peaks in control cells (Supplementary Fig. S5D). Strictly the same cut-offs were applied for each irradiated condition. An example for a 6 Gy irradiation at 4 MV is reported in Supplementary Fig. S6E.

### RT-qPCR (custom TLDA)

Seven days after irradiation, HUVECs were harvested with 600 µL per sample of mirVana miRNA Isolation Kit lysis buffer (THERMO FISHER SCIENTIFIC, AM1560). Total RNA was quantified on an ND-100 NanoDrop and samples were stored at −80 °C. Total RNA was diluted to 50 ng/µL (final concentration) and 500 ng was used to perform RT-PCR. cDNAs were loaded on customized TLDA. The PCR protocol was as follows: a preparation step (50 °C for two minutes followed by 10 min at 94.5 °C), then 40 cycles including denaturation (97 °C, 3 min), hybridization of primers and elongation (60 °C, 1 min). The Taqman Low Density Assay (TLDA) includes the following list of genes: Hs00909449_m1 (ACTA2), Hs00248075_m1 (BBC3), Hs00978503_m1 (BIRC5), Hs01075861_m1 (CD44), Hs00938777_m1 (CDK1), Hs00355782_m1 (CDKN1A), Hs00923894_m1 (CDKN2A), Hs00929873_m1 (CSF2), Hs99999083_m1 (CSF3), Hs00236330_m1 (FAS), Hs00169255_m1 (GADD45A), Hs00232622_m1 (HEY2), Hs01555410_m1 (IL1B), Hs00985639_m1 (IL6), Hs00174103_m1 (IL8), Hs00765730_m1 (NFKB1), Hs01126606_m1 (SERPINE1), Hs00998193_m1 (SMAD7), Hs00162558_m1 (TAGLN), Hs00936520_m1 (TP53I3), Hs00426287_m1 (IGFBP3), Hs00181213_m1 (IGFBP5), Hs00266026_m1 (IGFBP7), Hs01042796_m1 (MMP7), Hs00233987_m1 (MMP10), Hs00176481_m1 (CDKN2D), Hs00971716_m1 (CAV1), Hs01597588_m1 (CDKN1B), Hs00793225_m1 (CDKN2B), Hs00914223_m1 (EP300), Hs01059210_m1 (LMNB1), Hs01062014_m1 (NOTCH1), Hs01100061_m1 (PAK4), Hs01078066_m1 (RB1), Hs01009006_m1 (SIRT1), Hs00187842_m1 (B2M), Hs01119326_m1 (PTPRJ), Hs00190266_m1 (STX4), Hs00247916_m1 (LAT2), Hs00601975_m1 (CXCL2), Hs00174092_m1 (IL1A), Hs00989786_m1 (CEACAM1), Hs00174583_m1 (SELP), Hs00232219_m1 (SMAD3), Hs99999903_m1 (ACTB), Hs99999906_m1 (PGK1), Hs00177083_m1 (MAPK8) et Hs99999901_s1 (18 S). Analysis of data was performed using ExpressionSuite software (THERMO FISHER SCIENTIFIC), while representation and statistical analysis of the data were performed using DataAssist software (THERMO FISHER SCIENTIFIC).

### Statistical analysis

#### Clonogenic assay

The number of scored colonies *y*_*i*_(*d*) at each dose d and plate i, was modeled as a Bernoulli trial^39^:

$${y}_{i}(d) \sim {\mathfrak{B}}({N}_{i}(d)\,,\,S(d))$$

*N*_*i*_(*d*) is the number of seeded cells and $S(d)=PE\times \exp (-\alpha d-\beta {d}^{2})$ the “success” probability for a cell to grow into a colony. Here *PEα* and *β* are the model parameters, and *PE* represents the plating efficiency, i.e. the surviving fraction of non-irradiated cells. After inverting the fitted survival curves for each energy (200 kV and 4 MV), the relative biological effect (RBE) was computed as a ratio of physical doses that generate the same survival fraction and its associated confidence intervals were calculated by bootstrapping^40^.

#### Cell viability

Let *n*_*ij*_ designate the number of viable cells remaining *t*_*i*_ days after exposure to *d*_*j*_ Gy and *n*_*i*0_ the number of viable cells in the control sample at the same time point. We modeled the log ratio $L{R}_{ij}=log(\frac{{n}_{ij}}{{n}_{i0}})$ as a bivariate function of time *t*_*i*_ and dose *d*_*j*_ through the regression:

$$L{R}_{ij}={\beta }_{(4MV)}({t}_{i},\,{d}_{j})+{\chi }_{S(220kV)}\times {\beta }_{(4MV)vs(220kV)}({t}_{i},\,{d}_{j})+{\varepsilon }_{ij}$$

Where *β*_(4*MV*)_ and *β*_(4*MV*) *vs* (220 *kV*)*I*_ represent two bivariate penalized B-spline functions, ${\chi }_{(220kV)}$ is a dummy variable indicating cell irradiation by the 220 kV irradiator and *ε*_*ij*_ is the error term. Thus, by considering the 220 kV beam as reference, the comparison in time and dose between the viable cells with the two energies is driven by the function *β*_*I*_

$$\frac{Cell\,Viability\,(220\,kV)\,}{Cell\,Viability\,(4\,MV)}=exp({\beta }_{I}(t,\,d\,))$$

Computations for this study were carried out using the MATLAB Software, version 8.2.0.701 (Mathworks R2013b) and the REFUND package of R software.

#### Flow cytometry

One-way Anova using GraphPad Prism software was performed for all flow cytometry experiments (** p < 0.01, *** p < 0.001 and **** p < 0.0001).

#### RT-qPCR

RT-qPCR statistical analysis was automatically performed by ExpressionSuite and DataAssist software (corrected *p-value* by Benjamini-Hochberg False Discovery Rate test, p < 0.05).

## Supplementary information

Supplementary Table S1

Supplementary Information

## References

[1] Valentin J (2003). Relative biological effectiveness (RBE), quality factor (Q), and radiation weighting factor (wR):ICRP Publication 92: Approved by the Commission in January 2003. Annals of the ICRP.

[2] Puck TT, Marcus PI (1956). Action of x-rays on mammalian cells. The Journal of experimental medicine.

[3] Munshi A, Hobbs M, Meyn RE (2005). Clonogenic cell survival assay. Methods in molecular medicine.

[4] Franken NA, Rodermond HM, Stap J, Haveman J, van Bree C (2006). Clonogenic assay of cells *in vitro*. Nature protocols.

[5] Rafehi H (2011). Clonogenic assay: adherent cells. Journal of visualized experiments: JoVE.

[6] Chadwick, K. H. & Leenhouts, H. P. *The molecular theory of radiation biology /* XVII, 377 (Springer-Verlag Berlin Heidelberg, 1981).

[7] Kirkpatrick JP, Brenner DJ, Orton CG (2009). Point/Counterpoint. The linear-quadratic model is inappropriate to model high dose per fraction effects in radiosurgery. Medical physics.

[8] Abderrahmani R (2012). PAI-1-dependent endothelial cell death determines severity of radiation-induced intestinal injury. PloS one.

[9] Dos Santos, M. *et al*. Importance of dosimetry protocol for cell irradiation on a low X-rays facility and consequences for the biological response. *International journal of radiation biology*, 1–29, 10.1080/09553002.2018.1466205 (2018). 10.1080/09553002.2018.1466205 29701998

[10] Guipaud, O. *et al*. The importance of the vascular endothelial barrier in the immune-inflammatory response induced by radiotherapy. *The British journal of radiology*, 20170762, 10.1259/bjr.20170762 (2018). 10.1259/bjr.20170762 PMC6223160 29630386

[11] Korpela E, Liu SK (2014). Endothelial perturbations and therapeutic strategies in normal tissue radiation damage. Radiat Oncol.

[12] Gomolka M (2005). Measurement of the initial levels of DNA damage in human lymphocytes induced by 29 kV X rays (mammography X rays) relative to 220 kV X rays and gamma rays. Radiation research.

[13] Hill MA (2004). The variation in biological effectiveness of X-rays and gamma rays with energy. Radiation protection dosimetry.

[14] Kashino G (2004). Evidence for induction of DNA double strand breaks in the bystander response to targeted soft X-rays in CHO. cells. Mutation research.

[15] Slonina D (2003). Induction of micronuclei in human fibroblasts and keratinocytes by 25 kV x-rays. Radiation and environmental biophysics.

[16] Jones B (2017). Clinical radiobiology of proton therapy: modeling of RBE. Acta Oncol.

[17] Makita C (2014). Clinical outcomes and toxicity of proton beam therapy for advanced cholangiocarcinoma. Radiat Oncol.

[18] Wambersie A (1999). RBE, reference RBE and clinical RBE: applications of these concepts in hadron therapy. Strahlentherapie und Onkologie: Organ der Deutschen Rontgengesellschaft… [et al].

[19] Bjork-Eriksson T, West C, Karlsson E, Mercke C (2000). Tumor radiosensitivity (SF2) is a prognostic factor for local control in head and neck cancers. International journal of radiation oncology, biology, physics.

[20] Redon CE (2011). Recent developments in the use of gamma-H2AX as a quantitative DNA double-strand break biomarker. Aging.

[21] Tsamou M (2012). Performance of *in vitro* gammaH2AX assay in HepG2 cells to predict *in vivo* genotoxicity. Mutagenesis.

[22] Mori R, Matsuya Y, Yoshii Y, Date H (2018). Estimation of the radiation-induced DNA double-strand breaks number by considering cell cycle and absorbed dose per cell nucleus. Journal of radiation research.

[23] Chen X, Zhong Z, Xu Z, Chen L, Wang Y (2010). 2’,7’-Dichlorodihydrofluorescein as a fluorescent probe for reactive oxygen species measurement: Forty years of application and controversy. Free radical research.

[24] Morales A, Miranda M, Sanchez-Reyes A, Biete A, Fernandez-Checa JC (1998). Oxidative damage of mitochondrial and nuclear DNA induced by ionizing radiation in human hepatoblastoma cells. International journal of radiation oncology, biology, physics.

[25] Lafargue A (2017). Ionizing radiation induces long-term senescence in endothelial cells through mitochondrial respiratory complex II dysfunction and superoxide generation. *Free radical biology &*. medicine.

[26] Hernandez-Segura A, Nehme J, Demaria M (2018). Hallmarks of Cellular Senescence. Trends in cell biology.

[27] Pan J (2017). Inhibition of Bcl-2/xl With ABT-263 Selectively Kills Senescent Type II Pneumocytes and Reverses Persistent Pulmonary Fibrosis Induced by Ionizing Radiation in Mice. International journal of radiation oncology, biology, physics.

[28] Itahana K, Campisi J, Dimri GP (2007). Methods to detect biomarkers of cellular senescence: the senescence-associated beta-galactosidase assay. Methods Mol Biol.

[29] Debacq-Chainiaux F, Erusalimsky JD, Campisi J, Toussaint O (2009). Protocols to detect senescence-associated beta-galactosidase (SA-betagal) activity, a biomarker of senescent cells in culture and *in vivo*. Nature protocols.

[30] Dong X (2015). NEMO modulates radiation-induced endothelial senescence of human umbilical veins through NF-kappaB signal pathway. Radiation research.

[31] Campisi J (2007). & d’Adda di Fagagna, F. Cellular senescence: when bad things happen to good cells. Nature reviews. Molecular cell biology.

[32] Reyes J (2018). Fluctuations in p53 Signaling Allow Escape from Cell-Cycle Arrest. Molecular cell.

[33] Burton DG, Krizhanovsky V (2014). Physiological and pathological consequences of cellular senescence. Cellular and molecular life sciences: CMLS.

[34] Gruel G (2016). Cell to Cell Variability of Radiation-Induced Foci: Relation between Observed Damage and Energy Deposition. PloS one.

[35] Vaurijoux A, Voisin P, Freneau A, Barquinero JF, Gruel G (2017). Transmission of persistent ionizing radiation-induced foci through cell division in human primary cells. Mutation research.

[36] Canny J (1986). A computational approach to edge detection. IEEE transactions on pattern analysis and machine intelligence.

[37] Lobrich M (2010). gammaH2AX foci analysis for monitoring DNA double-strand break repair: strengths, limitations and optimization. Cell Cycle.

[38] Van Hooijdonk CA, Glade CP, Van Erp PE (1994). TO-PRO-3 iodide: a novel HeNe laser-excitable DNA stain as an alternative for propidium iodide in multiparameter flow cytometry. Cytometry.

[39] Shuryak I, Sun Y, Balajee AS (2016). Advantages of Binomial Likelihood Maximization for Analyzing and Modeling Cell Survival Curves. Radiation research.

[40] Good, P. I. *Permutation*, *Parametric*, *and Bootstrap Tests of Hypotheses*. 3 edn, XX, 316 (Springer New York, 2005).

