# Supplementary material for: Multiparametric radiobiological assays show that variation of X-ray energy strongly impacts relative biological effectiveness: comparison between 220 kV and 4 MV
Source: Sci Rep. 2019 Oct 4;9:14328. doi: 10.1038/s41598-019-50908-4 (PMC6778087; doi:10.1038/s41598-019-50908-4)
Supplement: Supplementary file 1 — Supplementary Table S1 [file 41598_2019_50908_MOESM1_ESM.pdf]

Multiparametric radiobiological assays show that variation of X-ray energy strongly impacts relative biological effectiveness: comparison between 220 kV and 4 MV

Vincent Paget, Mariam Ben Kacem, Morgane Dos Santos, Mohamed A. Benadjaoud, Frédéric Soysouvanh, Valérie Buard, Georges Tarlet, Aurélie Vaurijoux, Gaëtan Gruel, Agnès François, Olivier Guipaud and Fabien Milliat

Supplementary Table S1

SF	RBE (220 kV vs 4MV)	95% CI*
0.01	1.01	[0.97;1.06] #
0.02	1.03	[0.98;1.08] #
0.05	1.08	[1.02;1.14]
0.10	1.11	[1.06;1.17]
0.15	1.13	[1.07;1.20]
0.20	1.15	[1.08;1.22]
0.25	1.16	[1.08;1.25]
0.30	1.18	[1.09;1.28]
0.35	1.19	[1.09;1.31]
0.40	1.21	[1.09;1.34]
0.45	1.22	[1.09;1.37]
0.50	1.23	[1.09;1.40]
0.55	1.25	[1.09;1.43]
0.60	1.26	[1.09;1.46]
0.65	1.27	[1.09;1.49]
0.70	1.29	[1.09;1.52]
0.75	1.30	[1.10;1.55]
0.80	1.31	[1.10;1.58]
0.85	1.33	[1.10;1.62]
0.90	1.34	[1.10;1.66]
0.95	1.36	[1.10;1.69]

Supplementary Table S1: Clonogenic assay RBE values (Ratio of doses for a given SF). * 95% CI = 95% confidence interval. # Not significant (CI including 1).
