# Supplementary material for: Multiparametric radiobiological assays show that variation of X-ray energy strongly impacts relative biological effectiveness: comparison between 220 kV and 4 MV
Source: Sci Rep. 2019 Oct 4;9:14328. doi: 10.1038/s41598-019-50908-4 (PMC6778087; doi:10.1038/s41598-019-50908-4)
Supplement: Supplementary file 2 — Supplementary Information [file 41598_2019_50908_MOESM2_ESM.pdf]

Multiparametric radiobiological assays show that variation of X-ray energy strongly impacts relative biological effectiveness: comparison between 220 kV and 4 MV

Vincent Paget, Mariam Ben Kacem, Morgane Dos Santos, Mohamed A. Benadjaoud, Frédéric Soysouvanh, Valérie Buard, Georges Tarlet, Aurélie Vaurijoux, Gaëtan Gruel, Agnès François, Olivier Guipaud and Fabien Milliat

Supplementary Information

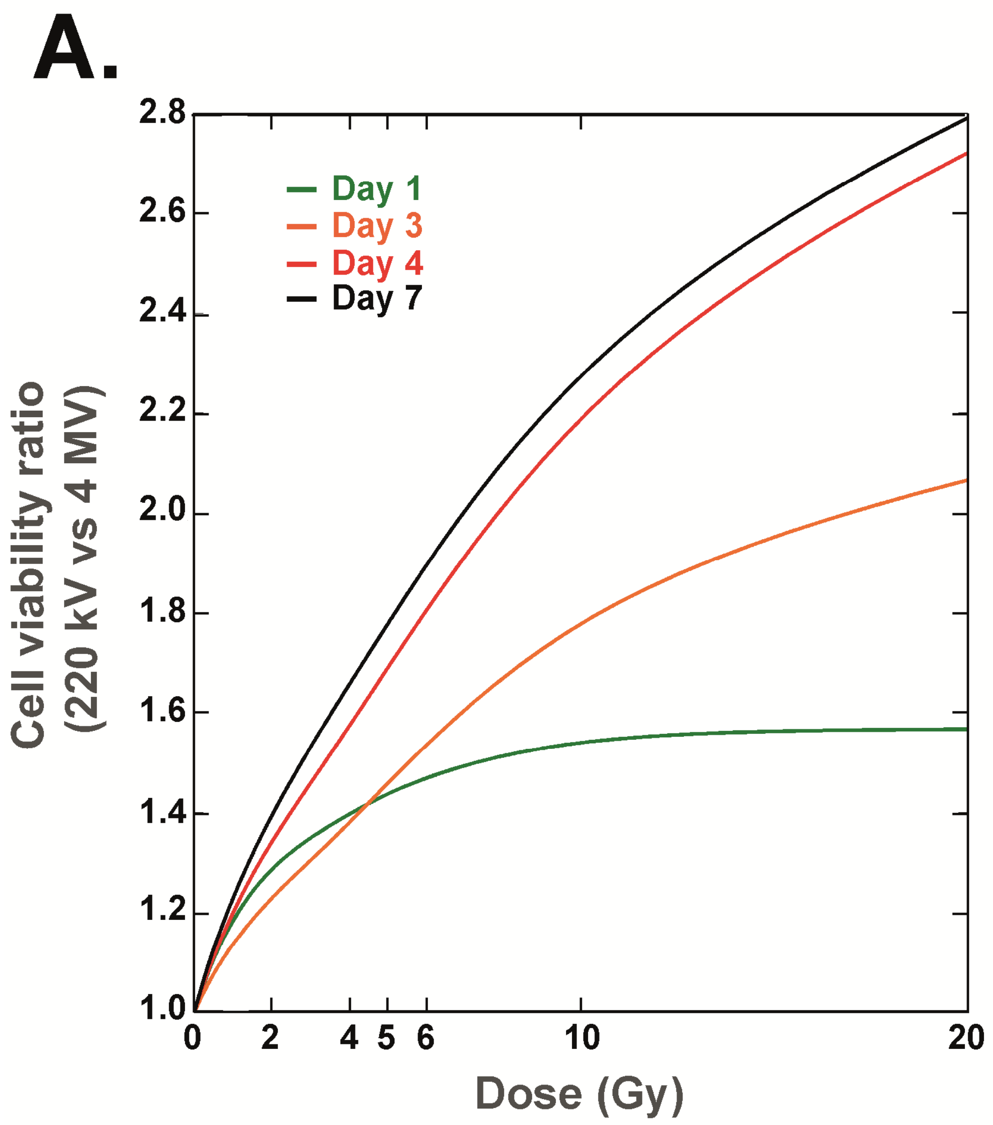

B.

Dose (Gy)	Time (Days)	RBE	95% CI*
2.00	1.00	1.28	[1.21;1.34]
2.00	2.00	1.33	[1.26;1.39]
2.00	3.00	1.22	[1.17;1.27]
2.00	4.00	1.33	[1.27;1.38]
2.00	7.00	1.38	[1.30;1.46]
4.00	1.00	1.38	[1.32;1.45]
4.00	2.00	1.49	[1.43;1.55]
4.00	3.00	1.36	[1.31;1.41]
4.00	4.00	1.54	[1.49;1.60]
4.00	7.00	1.62	[1.55;1.69]
5.00	1.00	1.43	[1.36;1.49]
5.00	2.00	1.57	[1.50;1.63]
5.00	3.00	1.43	[1.38;1.49]
5.00	4.00	1.65	[1.59;1.72]
5.00	7.00	1.74	[1.67;1.81]
6.00	1.00	1.47	[1.40;1.53]
6.00	2.00	1.65	[1.58;1.72]
6.00	3.00	1.53	[1.47;1.59]
6.00	4.00	1.79	[1.72;1.86]
6.00	7.00	1.88	[1.80;1.96]
10.00	1.00	1.54	[1.46;1.62]
10.00	2.00	1.84	[1.75;1.94]
10.00	3.00	1.76	[1.68;1.84]
10.00	4.00	2.16	[2.07;2.25]
10.00	7.00	2.24	[2.14;2.36]
20.00	1.00	1.57	[1.45;1.70]
20.00	2.00	2.05	[1.90;2.20]
20.00	3.00	2.07	[1.93;2.21]
20.00	4.00	2.72	[2.55;2.91]
20.00	7.00	2.79	[2.59;3.01]

Supplementary Figure S1. "RBE" based on cell viability (ratios for a given dose and time). (A) "RBE" representation at day 1 (green curve), day 3 (orange curve), day 4 (red curve) and day 7 (black curve) depending on the dose (Gy) (B) Numerical values of RBE extracted from experimental data. 95% CI = 95% confidence interval.

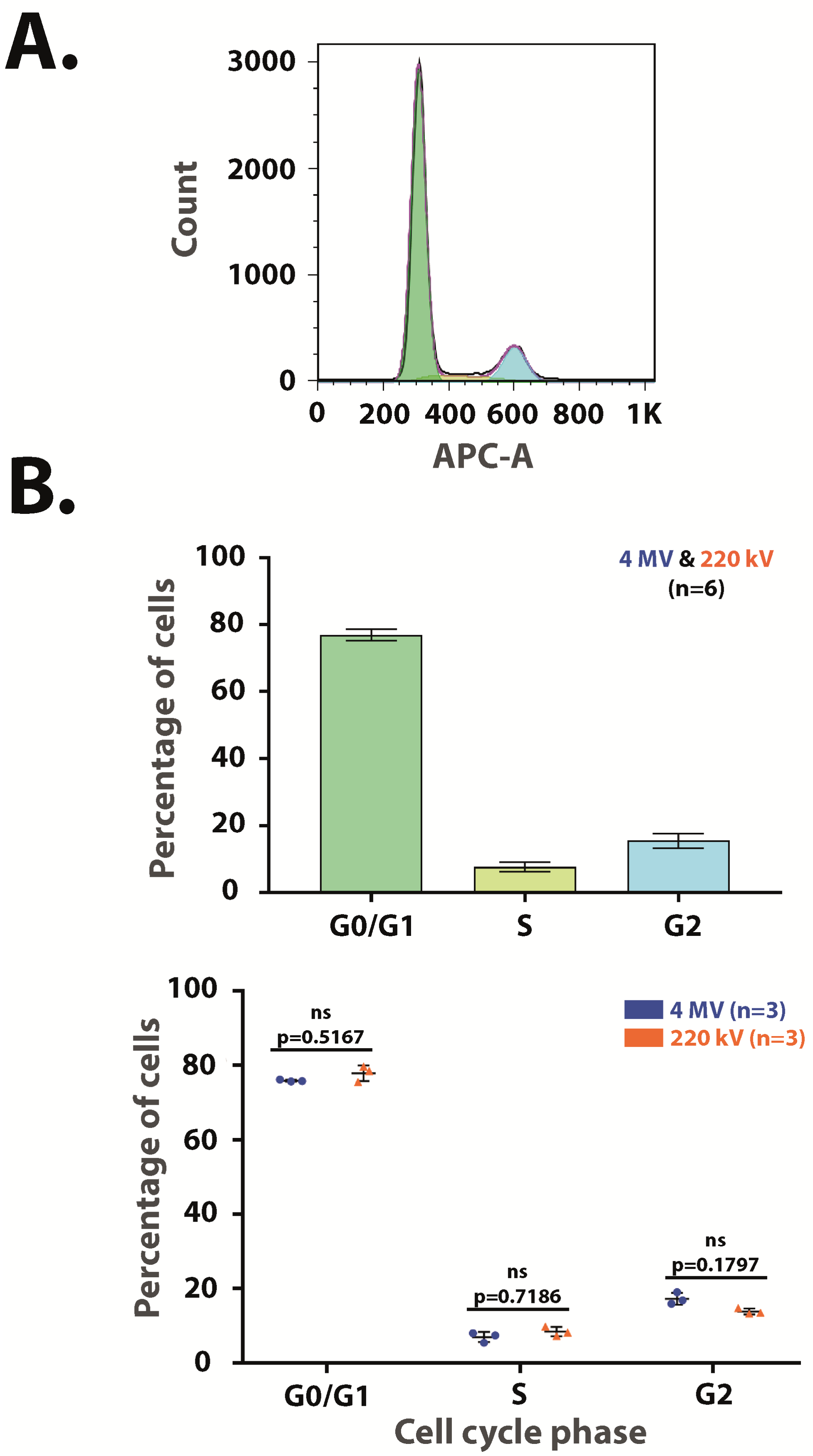

Supplementary Figure S2. Cell cycle analysis performed on controls just before irradiation. (A) Example of one representative cell cycle analysis performed using the FlowJo cell cycle tool on single cells (set-up to remove debris first, then by using size (APC-W)/intensity (APC-A) bi-parametric analysis to remove doublets) (B) Mean of cell cycle proportions for controls at T0 (n=6 total, upper panel); n=3 for 220 kV plus n=3 for 4 MV (bottom panel); $5x10^4$ cells were analyzed per n).

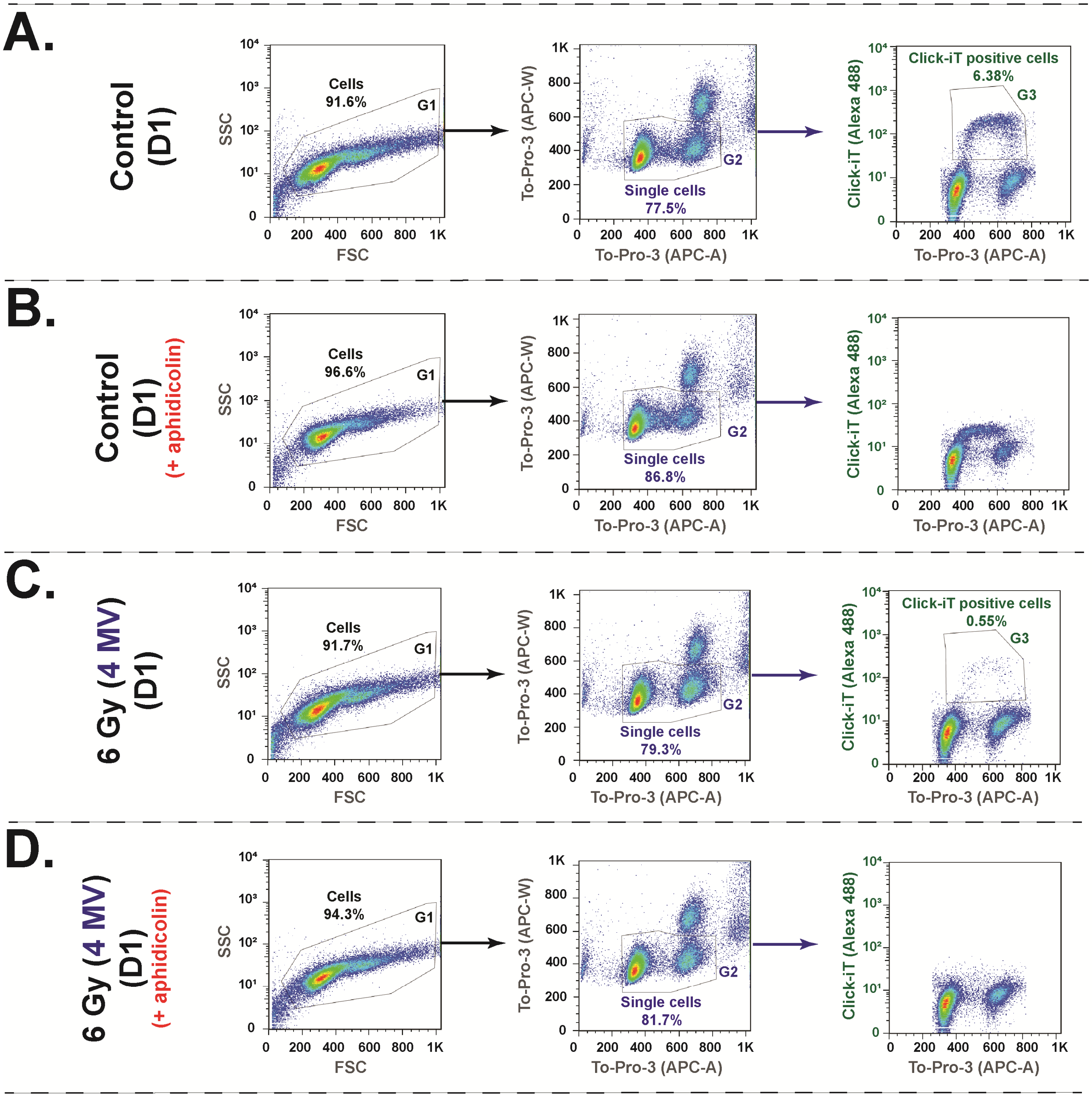

Supplementary Figure S3. Analysis set-up for Click-iT® EdU experiments. (A) Gating set-up to remove debris (gate G1) and to assess the correct detection of single events (gate G2) by using size (APC-W)/intensity (APC-A) bi-parametric analysis in negative control (DMSO) (B) Same analysis set-up allowing detection of positive Click-iT® positive cells (Gate G3) on the Alexa 488 channel (C and D) Represent rigorously same analysis set-up at day 1 for 6 Gy irradiation at 4 MV.

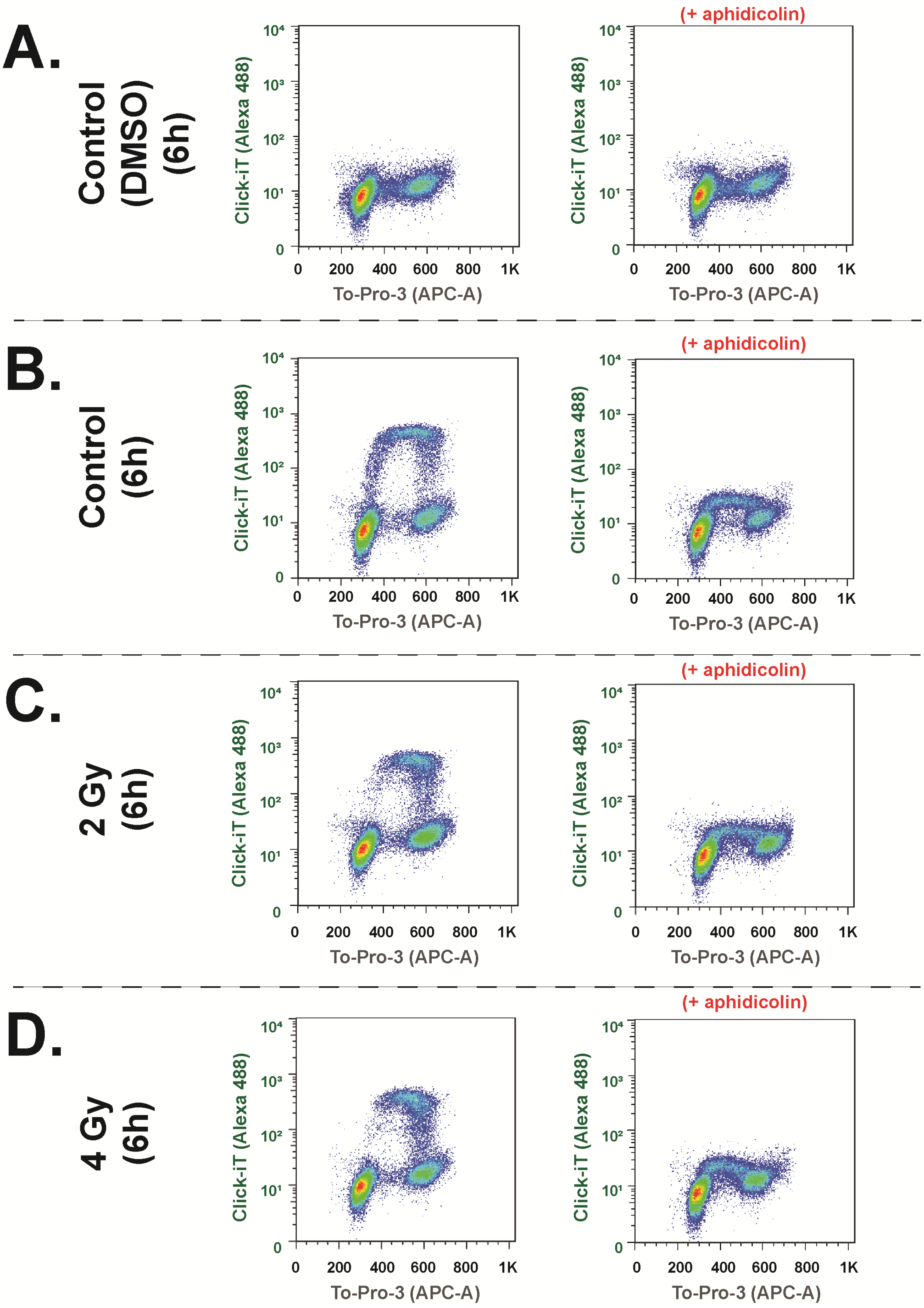
A.
Control (DMSO) (6h)
B.
Control (6h)
C.
2 Gy (6h)
D.
4 Gy (6h)
(+ aphidicolin)
Click-iT (Alexa 488)
To-Pro-3 (APC-A)
0
10¹
10²
10³
10⁴
200
400
600
800
1K

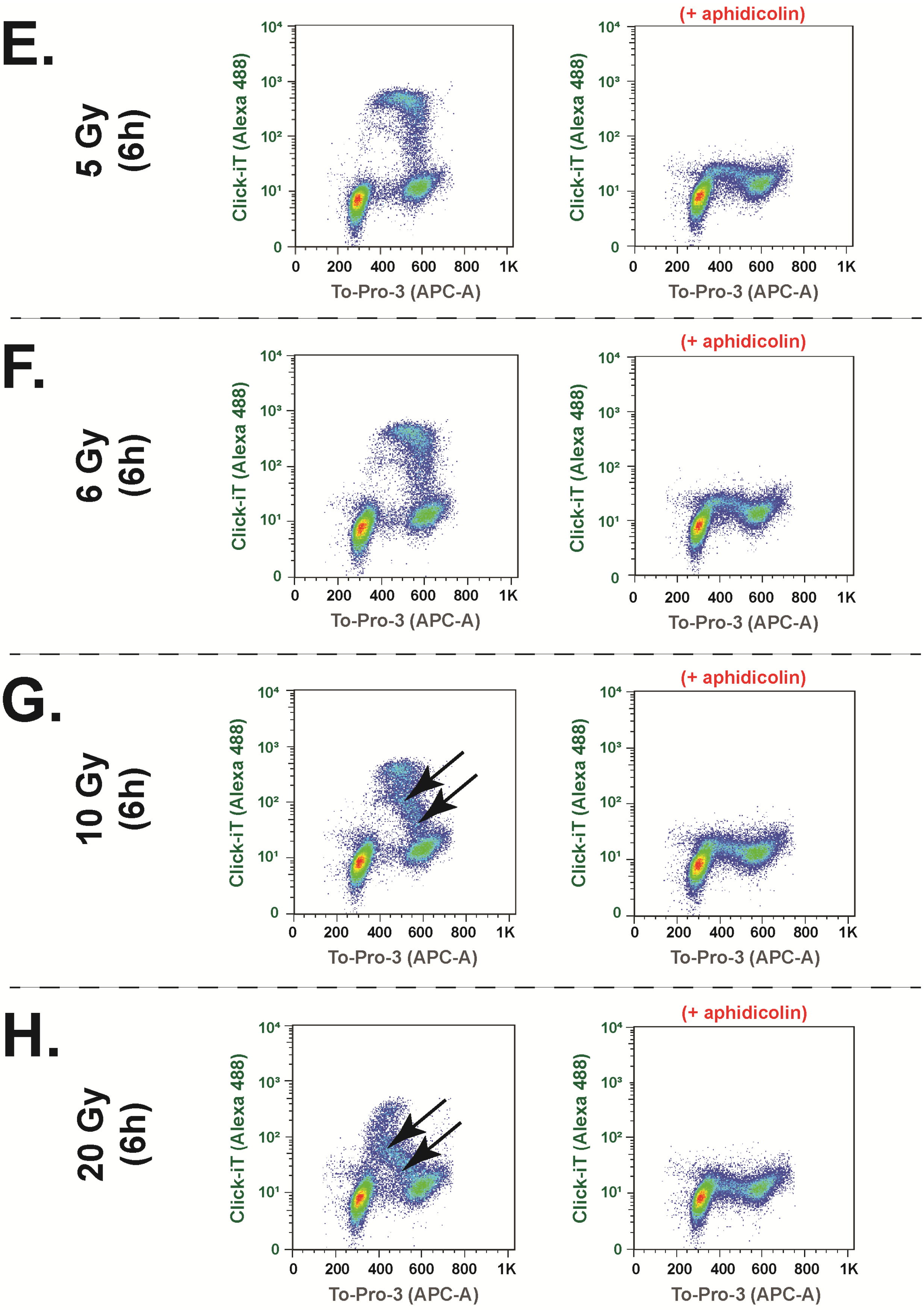

Supplementary Figure S4. Analysis of Click-iT® EdU experiments 6 hours after irradiation (4 MV). (A) Represents negative control with DMSO of Click-iT® detection for non-irradiated cells (B) Represents Click-iT® detection for non-irradiated cells (C to H) Represents Click-iT® detection for irradiated cells to 4 MV at 2, 4, 5, 6, 10 and 20 Gy, respectively. For each condition, right panel correspond to negative control for Click-iT® staining by the use of aphidicolin (A to H). Black arrows indicate altered incorporation of EdU (panels G and H).

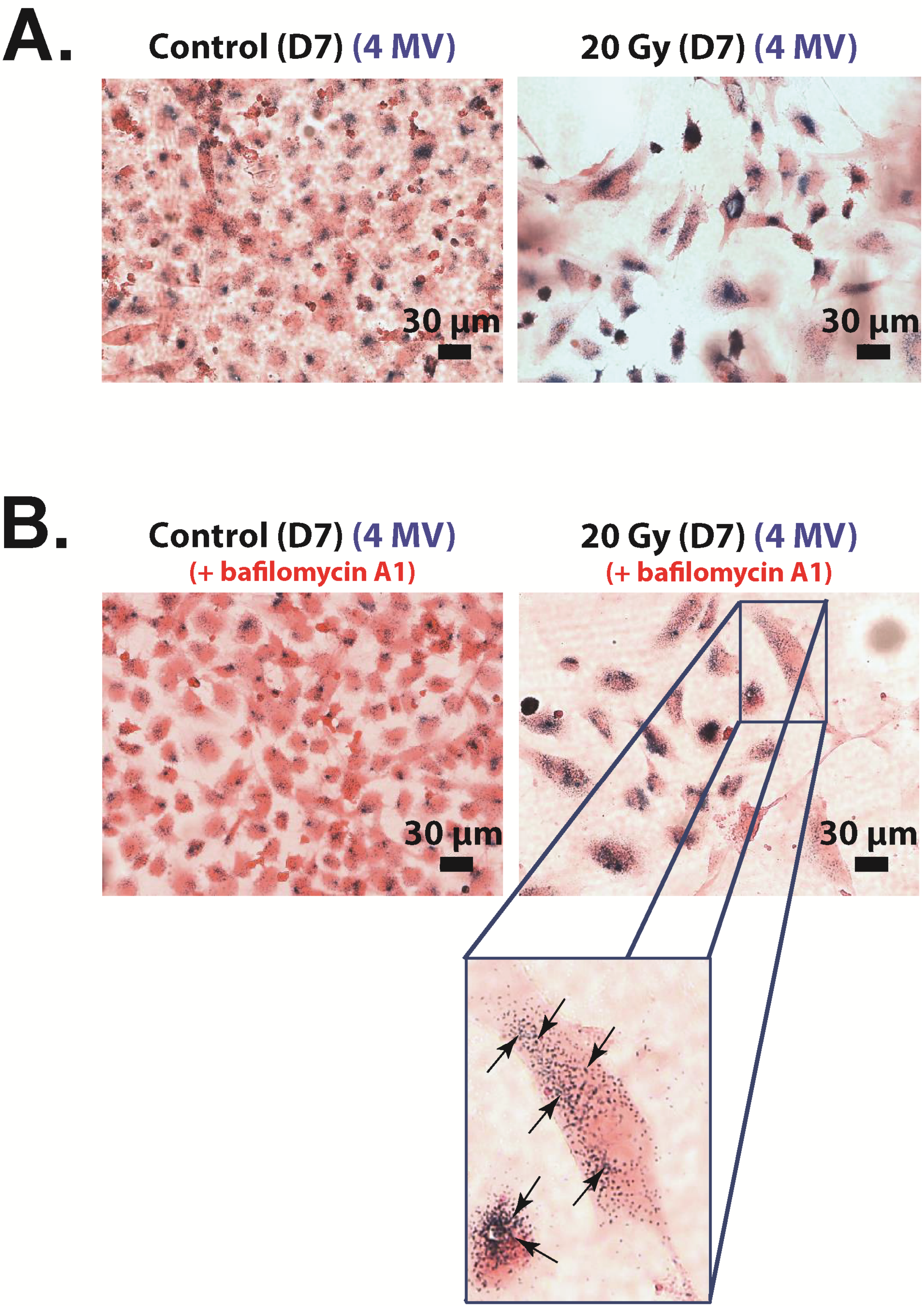

Supplementary Figure S5. X-GAL staining 7 days after irradiation by 20 Gy at 4 MV. (A) Represents staining obtained for conventional protocol (B) Represents staining obtained for modified protocol, consisting in 1-hour pre-treatment with bafilomycin A1. The dark arrows indicate X-GAL staining corresponding to lysosomes. Scale bar corresponds to 30 µm.

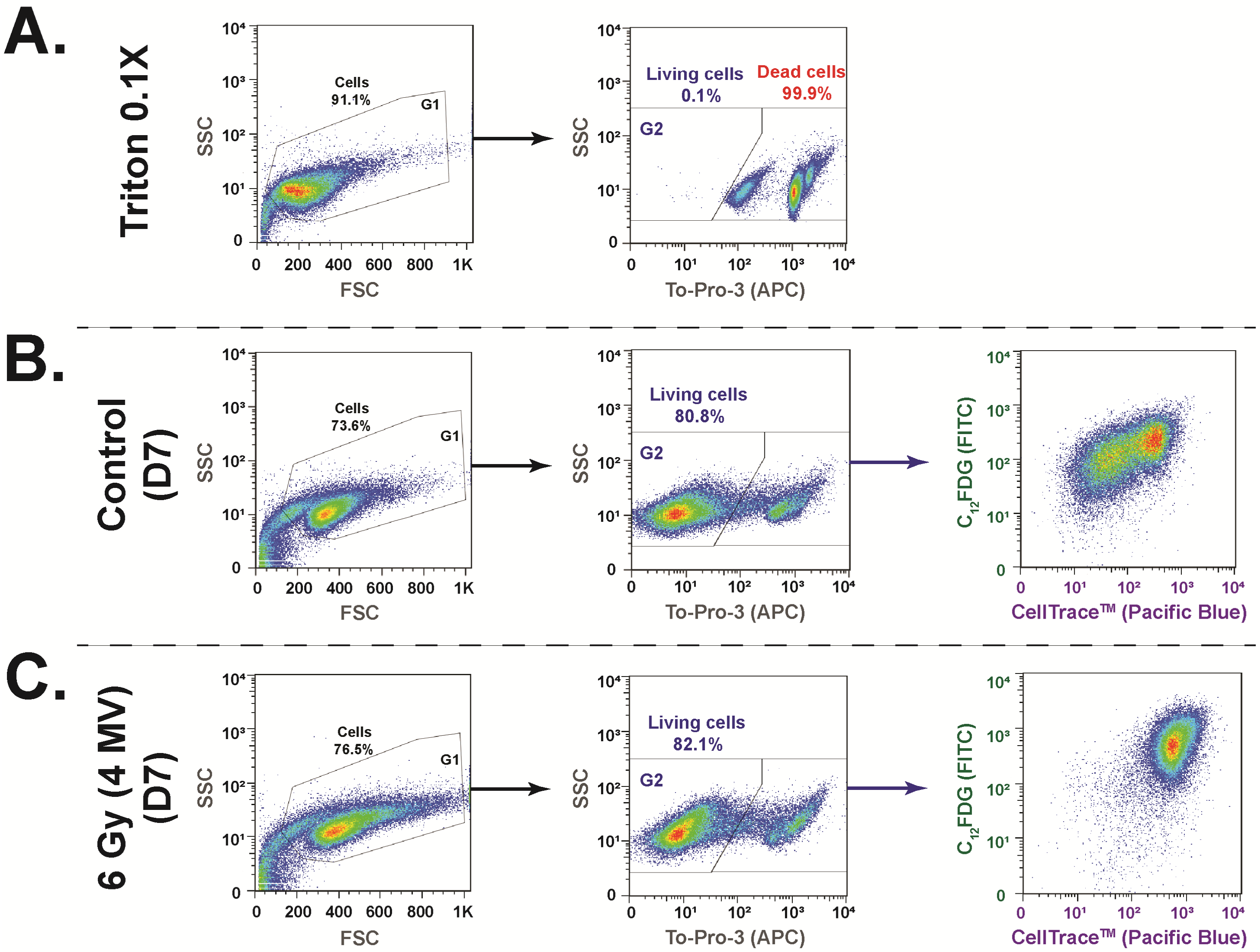

A.
Triton 0.1X
Cells
91.1%
G1
SSC
FSC
Living cells
0.1%
Dead cells
99.9%
G2
To-Pro-3 (APC)
B.
Control
(D7)
Cells
73.6%
Living cells
80.8%
C12FDG (FITC)
CellTrace™ (Pacific Blue)
C.
6 Gy (4 MV)
(D7)
Cells
76.5%
Living cells
82.1%

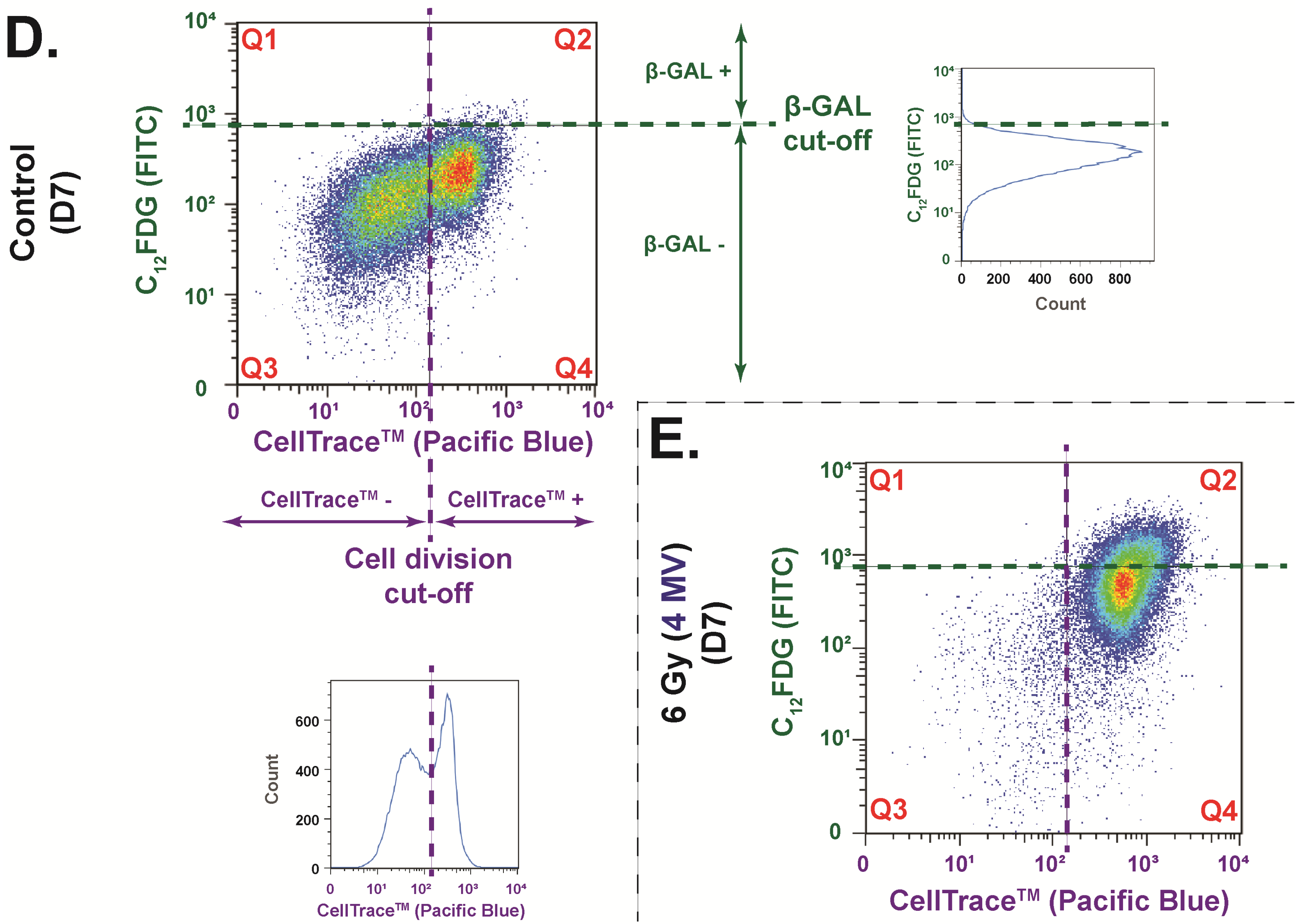

Supplementary Figure S6. Bi-parametric analysis set-up for C_{12}FDG/CellTrace. (A) Gating set-up to remove debris (gate G1) and to assess the correct detection on the APC channel of living (Gate G2) and dead cells by the use of positive control (Triton 0.X). (B) Bi-parametric representation of C_{12}FDG/CellTrace at day 7 post-irradiation for control condition (C) Bi-parametric representation of C_{12}FDG/CellTrace at day 7 after 6 Gy irradiation at 4 MV (D) cut-off settings set on control condition for C_{12}FDG and CellTrace (E) and applied to all other conditions, here at day 7 after 6 Gy irradiation at 4 MV for C_{12}FDG and CellTrace.

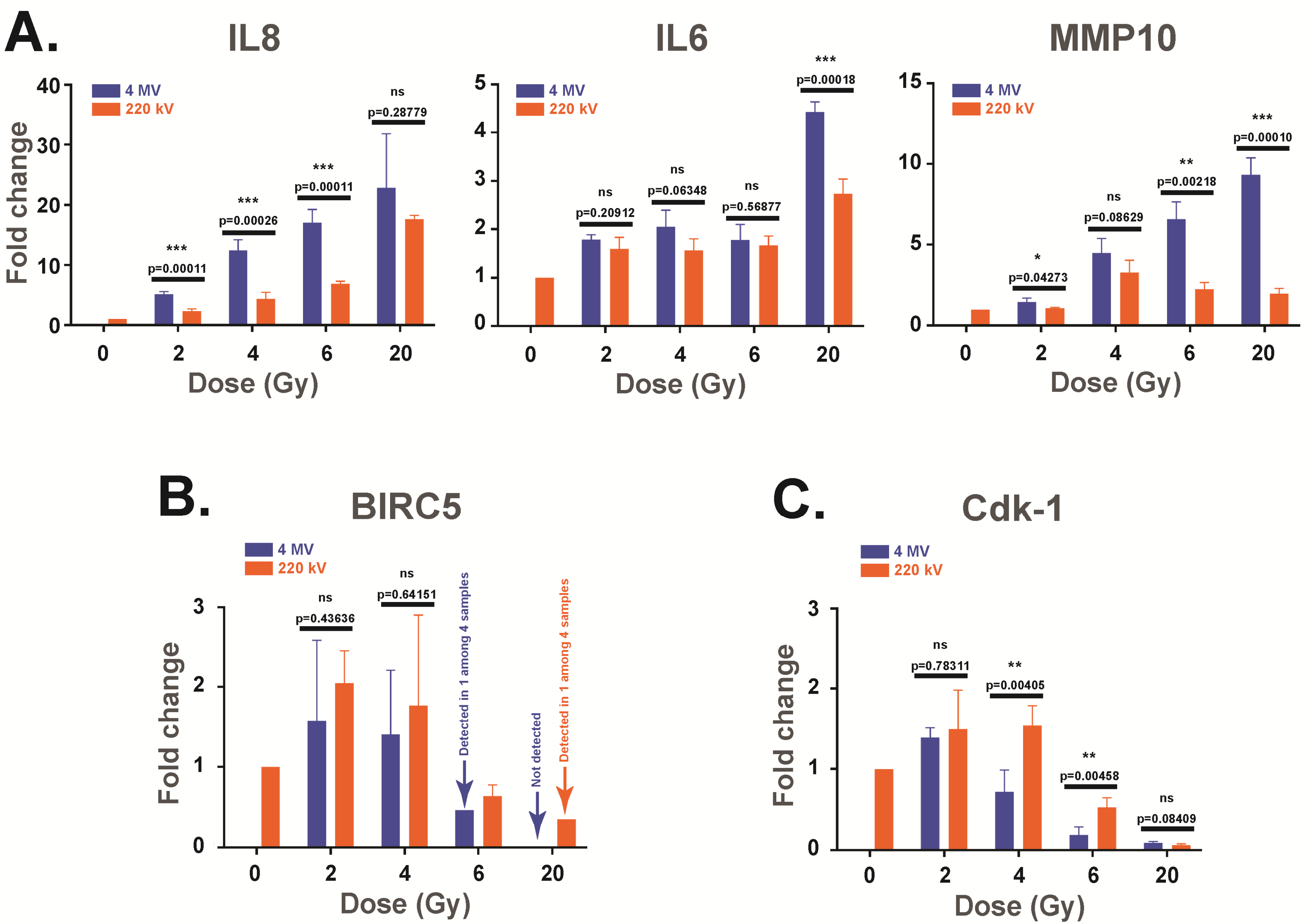

Supplementary Figure S7. IL-8, IL-6, MMP10, BIRC5 and Cdk-1 genes analysis from TLDA custom array at 7 days post-irradiation. Fold change compared to control at 4 MV (blue bars) and at 220 kV (orange bars) irradiations for IL-8 (A, left panel), IL-6 (A, middle panel), MMP10 (A, right panel), BIRC5 (B) and Cdk-1 (C) genes. Each bar represents the mean of 4 independent experiments (* $p < 0.05$, ** $p < 0.01$, *** $p < 0.001$, Student's t test), excepted for BIRC5 (B) where signal was not detected in all samples (for 6 and 20 Gy doses).
